# The mediating role of power distance and fear of negative evaluation in medical error under-reporting across surgical and internal divisions: a multicenter cross-sectional survey in Turkey

**DOI:** 10.1186/s12913-026-14895-3

**Published:** 2026-06-05

**Authors:** Melike Mercan Baspinar, Hamdiye Banu Katran, Ali Mirza Dincer, Feyzanur Erdem, Beray Gelmez Tas, Sundus Gorukmez, Resat Dabak, Guzin Zeren Ozturk, Okcan Basat, Secil Arica, Gokhan Adas

**Affiliations:** 1 Deputy Chief Medical Officer, University of Health Sciences, Gaziosmanpaşa Training and Research Hospital, Osmanbey Street, 621 Road, Gaziosmanpasa, Istanbul 34255 Turkey; 2 Department of Nursing Surgical Diseases Nursing, Marmara University Faculty of Health Sciences, Maltepe, Istanbul 34854 Turkey; 3 Department of Palliative Care Nursing, University of Health Sciences, Bakırkoy Sadi Konuk Training and Research Hospital, Bakırkoy, Istanbul 34153 Turkey; 4 Department of Family Medicine, University of Health Sciences, Prof. Dr. Cemil Tascioglu City Hospital, Darulaceze Street, Sisli, Istanbul, Turkey; 5 Department of Family Medicine, University of Health Sciences, Sisli Hamidiye Etfal Training and Research Hospital, Cumhuriyet ve Demokrasi Street, Sisli, Istanbul 34381 Turkey; 6 Department of Family Medicine, University of Health Sciences, Sultangazi Haseki Training and Research Hospital, Adnan Adıvar Street, Fatih, Istanbul 34096 Turkey; 7 Department of Family Medicine, University of Health Sciences, Gaziosmanpaşa Training and Research Hospital, Osmanbey Street, 621 Road, Gaziosmanpasa, Istanbul 34255 Turkey; 8 Department of General Surgery, University of Health Sciences, Prof. Dr. Cemil Tascioglu City Hospital, Darulaceze Street, Sisli, Istanbul Turkey

**Keywords:** Medical errors, Power distance, Fear, Patient safety, Organizational culture, Policy, Healthcare workforce, Turkey

## Abstract

**Background:**

Medical error under-reporting remains a critical barrier to patient safety, often driven by hierarchical organizational structures and fear-related mechanisms. This study aimed to examine the mediating role of fear of negative evaluation in the relationship between power distance and attitudes toward medical error reporting among healthcare professionals.

**Methods:**

A multicenter study of physicians and nurses was conducted in five tertiary hospitals in Istanbul, Turkey. Data were collected using anonymous, paper-based questionnaires with a stratified quota-based convenience sampling approach. The survey included validated scales assessing organizational power distance, fear of negative evaluation, and attitudes toward medical errors. Mediation analysis was conducted using regression-based models with bias-corrected and accelerated (BCa) bootstrap confidence intervals based on 5,000 resamples. Statistical significance was set at *p* < 0.05.

**Results:**

A total of 705 healthcare professionals participated (mean age: 32.3 [SD 8.5] years; 43.7% physicians, 56.3% nurses). More than half (51.9%) reported witnessing a medical error in the past year, whereas only 4.5% demonstrated positive attitudes toward error reporting. Internal medicine staff exhibited higher fear of negative evaluation and greater instrumental use of power compared with surgical staff (*p* < 0.01). Physicians reported higher fear of negative evaluation and greater use of instrumental power compared with nurses (*p* < 0.01). Fear of negative evaluation was positively associated with all power distance dimensions (*p* < 0.05). Mediation analysis showed that fear of negative evaluation mediates the relationship between instrumental use of power and medical error reporting attitudes (standardized β = 0.020, *p* = 0.013).

**Conclusion:**

Despite the high prevalence of witnessed medical errors, negative attitudes toward error reporting remained live. The findings suggest that hierarchical organizational dynamics and fear-related mechanisms may contribute to barriers to transparent reporting, highlighting the importance of psychologically safe and nonpunitive communication environments in hospital settings.

**Clinical trial number:**

Not applicable.

## Introduction

Power distance (PD) refers to the degree to which unequal power relations are accepted as normal features of organizational and institutional structures. Rooted in societal responses to human inequality, this concept is value-neutral and does not inherently imply positive or negative outcomes [1, 2]. PD-related challenges have emerged from aviation accident investigations, where hierarchical dynamics between superiors and subordinates have affected outcomes [2, 3]. While high-PD cultures trust authoritarian leadership, team members must voice concerns about flawed judgments. However, elevated PD can inhibit feedback, compromising safety and performance [1, 3, 4]. Employees often avoid direct communication with superiors in high PD settings [1]. Low PD cultures favor supportive leadership involving team participation [3]. In hospitals, staff participation and non-punitive error reporting can reduce hierarchical barriers [5]. High PD impedes communication and increases medical errors in diverse clinical settings [6].

The World Health Organization (WHO) defines patient safety as “the absence of preventable harm to a patient during the process of healthcare“ [7]. Medical errors are a leading cause of preventable harm globally [8]. According to the OECD, one in ten patients (10%) experiences harm during hospital care, causing over three million deaths yearly. In low- and middle-income countries, patient safety violations cause 134 million adverse events and 2.6 million deaths annually, making preventable harm a global health concern comparable to HIV/AIDS and traffic injuries [8, 9].

Accordingly, health managers must address the barriers to medical error reporting to advance patient safety. “To what extent does fear—particularly fear of negative evaluation—act as a barrier to medical error reporting among healthcare professionals?“. A systematic screening of 1,233 studies from 2015 to 2021 showed that fear-driven self-preservation and negative perceptions reduce error reporting. Psychologically safe environments encourage speaking up. These findings highlight the need for culturally sensitive interventions to promote transparent reporting practices [10]. Fear of negative consequences is the main barrier to incident reporting among nurses, along with system inadequacies, poor collaboration, lack of training, and blame culture [11].

To the best of our knowledge, no previous study has examined the relationship between fear of negative evaluation and medical error reporting among physicians and nurses within hierarchical organizational structures. This study explores how power distance (PD) and fear of negative evaluation influence under-reporting attitudes, and whether fear mediates the relationship between PD dimensions—acceptance, submission, legitimization, and instrumental use—and error reporting. It examines differences in fear, PD perception, and reporting attitudes across roles and departments among internal medicine and surgical divisions. Since fear of negative consequences—reinforced by hierarchy and blame culture—is a key barrier to reporting, this study contributes to understanding these dynamics.

## Method

### Design and setting

This multicenter survey study was conducted face-to-face among doctors and nurses in five tertiary-level training and research hospitals affiliated with the University of Health Sciences and coordinated by the Istanbul Provincial Health Directorate in Türkiye in 2022. A quantitative research approach was used to explore the relationships between power distance, fear of negative evaluation, and error reporting, and to test the potential mediating role of fear. Path analysis was applied to evaluate direct and indirect effects among variables. Regarding survey administration, data were collected using structured, paper-based self-administered questionnaires distributed on-site by trained research team members; participants completed the forms independently without interviewer assistance and returned them in sealed envelopes to ensure anonymity and minimize interviewer-related bias.

### Participants and sample size calculation

The study included physicians and nurses working in internal medicine or surgical departments who were actively involved in patient care, had at least one year of clinical experience, were aged ≥ 18 years, and provided informed consent. Healthcare personnel not directly involved in patient care, those on extended leave, individuals who declined participation, and questionnaires with substantial missing data in key variables were excluded. Participation was voluntary, and all eligible staff present during data collection were invited to participate.

An a priori sample size calculation based on small mediation effects (f² = 0.02) with up to six predictors (α = 0.05, power = 0.80) initially indicated a required sample of approximately 550 participants; however, a G*Power-based recalculation yielded a higher estimate (*n* ≈ 688). Participants were recruited on-site from predefined strata (physicians and nurses) proportionally across hospitals, and to account for nonresponse and incomplete questionnaires, a larger sample was targeted. The final analytic sample (*n* = 705) exceeded both estimated thresholds, ensuring adequate statistical power for the planned mediation and bootstrap analyses.

### Data collection measures

The study questionnaire form used in the study includes sociodemographic data and the items of three previously validated and published scales: Organisational power distance scale [12], Scale Of Attitudes Towards Medical Errors [13], Brief Fear of Negative Evaluation Scale [14].

#### Organisational power distance scale

Organisational power distance scale was measured using the Organisational Power Distance Scale developed by Yılmaz et al. The scale comprises 20 items across four subdimensions: *Acceptance of Power* (6 items), *Instrumental Use of Power* (5 items), *Legitimization of Power* (3 items), and *Submission to Power* (6 items). Items are rated on a 5-point Likert scale (1 = Never to 5 = Always), with three reverse-coded items. Exploratory and confirmatory factor analyses supported the four-factor structure, explaining 56.6% of total variance, with acceptable-to-good model fit indices. Internal consistency was satisfactory, with Cronbach’s alpha values ranging from 0.74 to 0.80. The scale does not generate a total score; instead, subscale scores are interpreted independently, where higher scores indicate stronger endorsement of the corresponding power distance dimension, enabling a differentiated assessment of hierarchical power dynamics within organizations [12]. **Legitimization of power (LP)** rationalizes unequal power distribution in organizations through legal rules, authority, and cultural beliefs, making it accepted by employees. This normalizes unequal practices and privileges [12, 15]. **Instrumental use of power** denotes using power to achieve goals at the expense of ethical principles [14, 16]. Employees may believe that aligning with superiors facilitates career advancement. This instrumentalization represents an ethical concern in bureaucratic systems [14, 17]. **Acceptance of power** reflects the internalization of legitimized power as natural and appropriate. This dimension shows how individuals in lower positions defer to superiors’ decisions [14, 18, 19], reinforcing hierarchical structures [14, 20]. **Submission to power** reflects compliance driven by fear or belief in unchangeable power relations, representing passive adaptation to authority [14]. Drawing from Gramsci, it shows alignment with power structures without internalized legitimacy [14, 21].

#### Scale of attitudes towards medical errors

The scale which was developed by Güleç and Seren-İntepeler consists of 16 items rated on a 5-point Likert scale (1 = Strongly disagree to 5 = Strongly agree) and comprises three subdimensions: Perception of Medical Errors, Approach to Medical Errors, and Causes of Medical Errors. Two items are reverse-coded. Exploratory and confirmatory factor analyses supported the three-factor structure, and the scale demonstrated acceptable model fit. Internal consistency was satisfactory, with a Cronbach’s alpha of 0.75 for the total scale, while test–retest reliability was high (*r* = 0.91), indicating temporal stability. Scale scores are calculated by averaging item responses, with higher scores reflecting more positive attitudes toward medical errors and error reporting; a cut-off value of 3 distinguishes negative from positive attitudes [13].

#### Brief fear of negative evaluation scale

The scale, developed by Leary and validated to Turkish by Çetin et al. The scale consists of 12 items rated on a 5-point Likert scale (1 = Not at all characteristic of me to 5 = Extremely characteristic of me), with reverse-coded items. It measures individuals’ apprehension about others’ evaluations, expectations of negative judgment, and concerns about social rejection. The scale has demonstrated strong psychometric properties, including high internal consistency (Cronbach’s α reported in previous studies > 0.80) and test–retest reliability of 0.82. Higher total scores indicate greater fear of negative evaluation, reflecting increased sensitivity to social judgment in professional contexts [14].

#### Sociodemographic data

The questions concern the employees’ age, gender, marital status, profession, educational background, department they work in, working style, and weekly working hours.

### Data analysis

Data analyses were conducted using jamovi (version 2.7.11) and SPSS 25.0 software. Continuous variables were summarised as mean ± standard deviation (SD), and categorical variables as number (n) and percentage (%). Group comparisons between two categories (e.g., profession, department, witnessing a medical error) were performed using the independent samples t-test, while comparisons among three or more groups (e.g., age categories, years of professional experience, monthly on-call duties, work schedule, and region of childhood) were conducted using one-way analysis of variance (ANOVA). When the overall ANOVA was statistically significant, post-hoc multiple comparisons were performed. Statistical significance was set at *p* < 0.05 (two-tailed).

To examine the hypothesised mediation model, regression-based mediation analyses were performed using jamovi. Acceptance of power (AP), submission to power (SP), legitimization of power (LP), and instrumental use of power (UP) were entered as independent variables, fear of negative evaluation as the mediator, and attitudes toward medical error reporting as the outcome variable. Bootstrap resampling with 5,000 iterations was applied to estimate indirect effects and corresponding confidence intervals. Effect sizes were reported as completely standardised coefficients (β) with standard errors, *z* values, and 95% confidence intervals. Mediation was considered statistically significant when the bootstrap confidence interval for the indirect effect did not include zero. No AI tools were used in any stage of data collection, data entry, statistical analysis, or interpretation of results; AI-assisted tools were restricted to grammar checking and literature organisation as detailed in the AI Disclosure Statement.

## Results

### Respondent characteristics

A total of 705 healthcare professionals participated in the study, with a mean age of 32.26 (8.47) years. Physicians constituted 43.7% of the sample (*n* = 308), while nurses accounted for 56.3% (*n* = 397). As presented in Table 1, more than half of the participants were married (57.9%) and worked in internal medicine departments (65.2%). Approximately half of the respondents (50.2%) had ≤ 5 years of professional experience, and 31.9% reported six or more monthly on-call duties. Nearly half of the participants (51.9%) reported witnessing a physician- or nurse-related medical error in the past year; however, only 4.5% demonstrated a positive attitude toward medical error reporting.

**Table 1:** Sociodemographic variables in the study

Variables	Groups	*n*	%
Age (year)	≤ 25	136	19.3
	26–35	370	52.6
	36–45	140	19.9
	≥ 46	58	8.2
Profession	Physician	308	43.7
	Nurse	397	56.3
Marital status	Single/Divorse	297	42.1
	Married	408	57.9
Division	Internal Division	460	65.2
	Surgery Division	245	34.8
Job experience (year)	≤ 5	354	50.2
	6–10	161	22.8
	≥ 11	190	27.0
Montly shift number	None	244	34.6
	1–5	236	33.5
	≥ 6	225	31.9
Working programme	Daytime^a^	300	42.6
	Night^b^	41	5.8
	Mostly during the day^c^	218	30.9
	Mostly at night^d^	146	20.7
Witnessing a medical error in the last 1 year	Yes	366	51.9
	No	339	48.1
Attitude on reporting	Negative attitude	673	95.5
	Positive attitude	32	4.5
Region of childhood	Marmara region	233	33.0
	Black Sea region	66	9.4
	Eastern Anatolia region	64	9.1
	Central Anatolia region	100	14.2
	Southeast Anatolia region	79	11.2
	Mediterranean region	87	12.3
	Aegean region	76	10.8

### Differences in attitudes toward medical errors and power distance dimensions

Significant differences were identified across several sociodemographic and occupational variables (Table 2). Fear of negative evaluation varied significantly by age group, with higher scores observed among participants aged ≤ 25 years compared with those aged 36–45 years (*p* = 0.006). Legitimization of power was higher among participants aged ≥ 46 years than those aged 26–35 years (*p* = 0.025).

**Table 2:** Comparison of attitudes toward medical errors and power dynamics across sociodemographic variables

		Attitude Toward Medical Error	Fear of Negative Evaluation	Acceptance of Power	Submission to Power	Legitimization of Power	Instrumental Use of Power
		Mean ± SD	Mean ± SD	Mean ± SD	Mean ± SD	Mean ± SD	Mean ± SD
Age	<= 25 (1)	2.35 ± 0.41	31.74 ± 8.37	17.42 ± 3.75	12.64 ± 4	5.95 ± 2.3	16.59 ± 4.85
	26–35 (2)	2.3 ± 0.37	30.93 ± 9.46	17.22 ± 3.94	12.62 ± 4.04	5.65 ± 2.11	16.53 ± 4.83
	36–45 (3)	2.3 ± 0.37	28.55 ± 8.78	17.84 ± 4.14	12.4 ± 4.23	5.85 ± 2.2	16.29 ± 4.87
	46+ (4)	2.34 ± 0.33	28.55 ± 9.01	18.1 ± 4.63	12.19 ± 4.73	6.69 ± 2.53	16.53 ± 4.43
F value		0.934	**4.144**	1.355	0.267	**3.196**	0.113
p value		0.424	0.006*	0.255	0.849	0.025*	0.953
Post-hoc			**1 > 3**			**4 > 2**	
Profession	Physician	2.36 ± 0.33	31.65 ± 9.62	17.3 ± 3.87	13.45 ± 3.97	5.93 ± 2.12	17.21 ± 4.66
	Nurse	2.28 ± 0.4	29.47 ± 8.65	17.58 ± 4.11	11.84 ± 4.1	5.76 ± 2.29	15.92 ± 4.84
t value		**2.968**	**3.114**	-0.949	**5.251**	0.974	**3.567**
p value		0.003*	0.002*	0.343	0.000*	0.330	0.000*
Marital status	Married	2.33 ± 0.37	30.36 ± 9.23	17.92 ± 4.05	12.81 ± 4.08	6.06 ± 2.25	16.87 ± 4.67
	Single	2.3 ± 0.38	30.46 ± 9.1	17.12 ± 3.95	12.35 ± 4.15	5.67 ± 2.17	16.21 ± 4.88
t value		0.901	-0.139	**2.636**	1.460	**2.292**	1.815
p value		0.368	0.889	0.009*	0.145	0.022*	0.070
Department	Internal Medicine	2.33 ± 0.36	31.34 ± 9.37	17.41 ± 4	12.55 ± 4.05	5.76 ± 2.11	16.92 ± 4.66
	Surgical Sciences	2.28 ± 0.39	28.69 ± 8.45	17.56 ± 4.04	12.55 ± 4.27	5.97 ± 2.39	15.68 ± 4.97
t value		1.551	**3.808**	-0.468	-0.004	-1.125	**3.277**
p value		0.121	0.000*	0.640	0.997	0.261	0.001*
Years of Professional Experience	<= 5 (1)	2.31 ± 0.36	30.77 ± 9.26	17.19 ± 3.63	12.49 ± 4.09	5.64 ± 2.11	16.41 ± 4.87
	6—10 (2)	2.35 ± 0.43	31.42 ± 8.74	17.55 ± 4.41	12.71 ± 3.83	5.93 ± 2.27	16.82 ± 4.58
	11+ (3)	2.29 ± 0.34	28.92 ± 9.12	17.88 ± 4.29	12.5 ± 4.43	6.12 ± 2.32	16.35 ± 4.87
F value		1.068	**3.799**	1.845	0.173	**3.200**	0.509
p value		0.345	0.023*	0.160	0.841	0.041*	0.601
Post-hoc			**2 > 3**			**3 > 1**	
Average Monthly Number of On-Call Duties	None (1)	2.31 ± 0.36	30.25 ± 9.55	17.4 ± 4.3	12.7 ± 4.17	5.86 ± 2.09	16.89 ± 4.99
	1—5 (2)	2.34 ± 0.39	32.06 ± 9.05	17.68 ± 3.73	13.08 ± 4.02	6.03 ± 2.35	16.87 ± 4.81
	6+ (3)	2.29 ± 0.36	28.89 ± 8.52	17.28 ± 3.98	11.81 ± 4.08	5.6 ± 2.18	15.66 ± 4.49
F value		0.948	**7.112**	0.604	**5.875**	2.173	**4.997**
p value		0.388	0.001*	0.547	0.003*	0.115	0.007*
Post-hoc			**2 > 3**		**2 > 3**		**1.2 > 3**
Work Schedule	Permanent day shift (1)	2.3 ± 0.36	30.85 ± 9.72	17.89 ± 4.22	12.69 ± 4.09	5.9 ± 2.12	17.06 ± 4.88
	Permanent night shift (2)	2.37 ± 0.45	28.98 ± 8	17.93 ± 3.54	12.02 ± 4.09	5.29 ± 1.91	15.05 ± 4.35
	Mostly day shift (3)	2.32 ± 0.37	30.91 ± 8.64	17.42 ± 3.87	13.03 ± 4.1	5.84 ± 2.24	16.52 ± 4.63
	Mostly night shift (4)	2.3 ± 0.39	29.21 ± 8.89	16.5 ± 3.75	11.67 ± 4.11	5.84 ± 2.43	15.67 ± 4.85
F value		0.588	1.636	**4.173**	**3.583**	0.908	**4.113**
p value		0.623	0.180	0.006*	0.014*	0.437	0.007*
Post-hoc				**1 > 4**	**1 > 4**		**1 > 4**
Witnessed a Physician- or Nurse-Related Medical Error in the Last Year	Yes	2.28 ± 0.37	30.87 ± 8.97	17.14 ± 4.17	12.7 ± 4.22	5.79 ± 2.23	16.46 ± 4.85
	No	2.35 ± 0.37	29.93 ± 9.32	17.8 ± 3.81	12.38 ± 4.01	5.88 ± 2.2	16.52 ± 4.75
t value		**-2.714**	1.364	**-2.189**	1.008	-0.520	-0.150
p value		0.007*	0.173	0.029*	0.314	0.603	0.880
Geographical Region of Childhood	Marmara (1)	2.28 ± 0.37	30.24 ± 9.52	17.9 ± 3.9	12.74 ± 4.36	5.94 ± 2.19	16.93 ± 4.84
	Black Sea (2)	2.26 ± 0.4	32.74 ± 8.49	18.08 ± 3.93	12.39 ± 3.91	5.83 ± 2.06	16.8 ± 4.53
	Eastern Anatolia (3)	2.36 ± 0.4	30.63 ± 8.38	17.61 ± 4.2	12.75 ± 4.11	6.14 ± 2.36	15.86 ± 4.86
	Central Anatolia (4)	2.3 ± 0.31	31.43 ± 9.8	17.54 ± 4.22	12.64 ± 4.66	5.56 ± 2.25	15.98 ± 5.04
	Southeastern Anatolia (5)	2.43 ± 0.38	29.27 ± 7.71	16.3 ± 3.61	12.18 ± 3.81	5.51 ± 2.18	16.84 ± 5.01
	Mediterranean (6)	2.3 ± 0.39	29.53 ± 9.49	16.68 ± 4.1	12.07 ± 3.59	6 ± 2.44	15.48 ± 4.9
	Aegean (7)	2.31 ± 0.38	29.66 ± 9.03	17.43 ± 4.03	12.71 ± 3.73	5.75 ± 2.01	16.84 ± 4.05
F value		1.916	1.372	**2.420**	0.518	0.945	1.528
p value		0.076	0.223	0.025*	0.794	0.462	0.166
Post-hoc				**1 > 5**			

F value: One-way Analysis of Variance (ANOVA); t: Independent samples t-test *p < 0.05

Physicians reported significantly higher scores than nurses in attitudes toward medical errors, fear of negative evaluation, and two PD dimensions: submission to power, and instrumental use of power (all *p* < 0.01). Married participants demonstrated higher acceptance of power and legitimization of power compared with single participants (*p* = 0.009 and *p* = 0.022, respectively).

Participants working in internal medicine departments had significantly higher fear of negative evaluation and instrumental use of power compared with those in surgical departments (*p* < 0.01). According to years of professional experience, fear of negative evaluation was higher among those with 6–10 years of experience compared with those with ≥ 11 years, while legitimization of power was higher among participants with ≥ 11 years of experience than those with ≤ 5 years (*p* < 0.05).

Monthly workload was also associated with significant differences. Participants with 1–5 monthly on-call duties reported higher fear of negative evaluation and submission to power than those with ≥ 6 duties, whereas participants with ≥ 6 monthly duties demonstrated lower instrumental use of power scores compared with the other workload groups (*p* < 0.01).

Work schedule was significantly associated with acceptance of power, submission to power, and instrumental use of power, with higher scores observed among permanent day-shift workers compared with mostly night-shift workers (*p* < 0.05). Participants who had witnessed a medical error within the last year reported lower attitudes toward medical errors and lower acceptance of power than those who had not (*p* < 0.05). Finally, acceptance of power differed significantly according to geographical region of childhood, with higher scores among participants from the Marmara region compared with those from Southeastern Anatolia (*p* = 0.025).

### Correlation analysis of power distance dimensions and fear of negative evaluation and attitudes toward medical errors

**Fig. 1:**
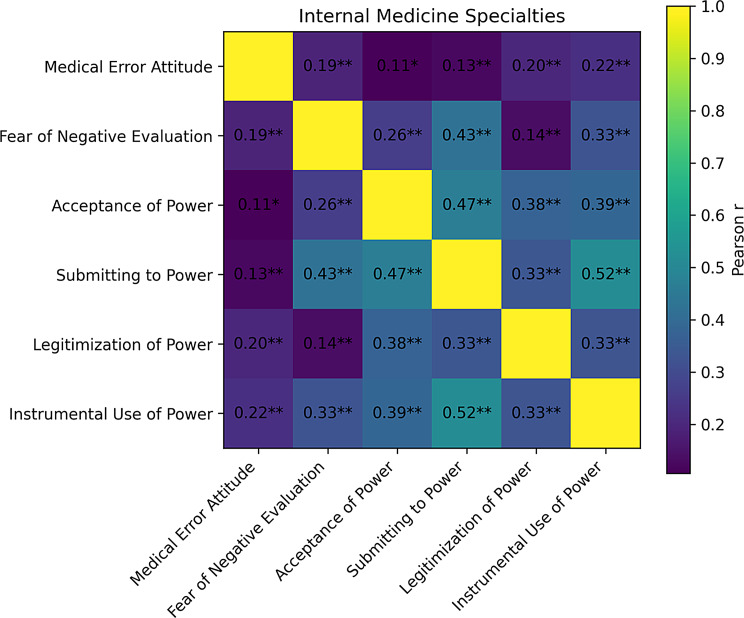
Correlation heat map showing associations between attitudes toward medical error reporting, fear of negative evaluation, and power distance dimensions in internal medicine divisions. Correlation coefficients are presented as Pearson’s r values. Asterisks indicate statistical significance: *p < 0.05; **p < 0.01

**Fig. 2:**
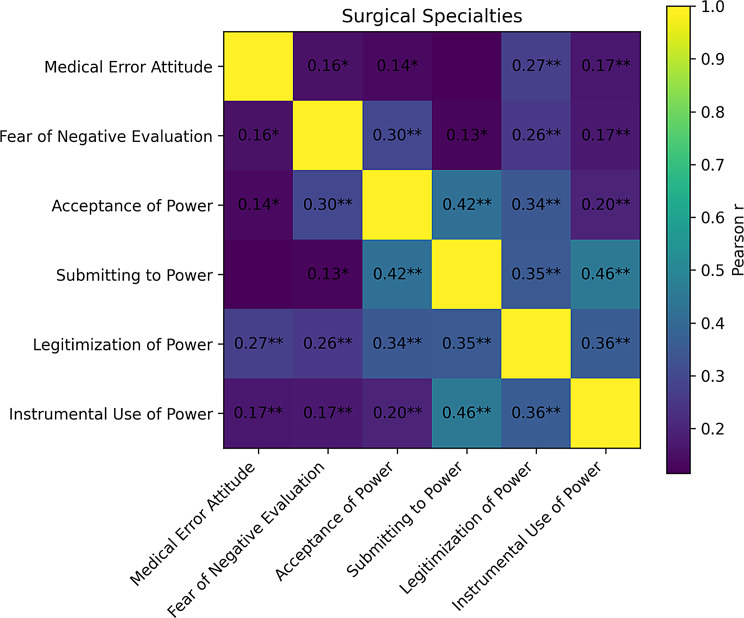
Correlation heat map showing associations between attitudes toward medical error reporting, fear of negative evaluation, and power distance dimensions in surgical divisions. Correlation coefficients are presented as Pearson’s r values. Asterisks indicate statistical significance: *p < 0.05;**p < 0.01

**Fig. 3:**
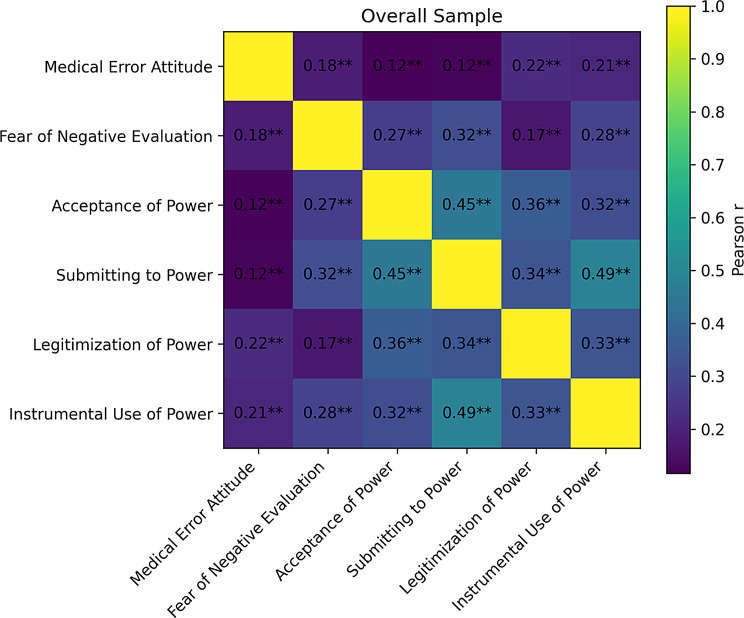
Correlation heat map showing associations between attitudes toward medical error reporting, fear of negative evaluation, and power distance dimensions in the overall study sample. Correlation coefficients are presented as Pearson’s r values. Asterisks indicate statistical significance: *p < 0.05; **p < 0.01

As shown in Table 3 and Figs. 1, 2 and 3, medical error reporting attitudes were consistently and positively associated with fear of negative evaluation and power distance dimensions across internal medicine, surgical specialties, and the overall sample (all *p* < 0.05).

**Table 3:** Correlations between attitude toward medical error, fear of negative evaluation, and power distance dimensions by specialty (internal medicine, surgical sciences, and overall sample)

			1	2	3	4	5	6
Internal Medicine	1) Attitude Toward Medical Error	r	1					
		p						
	2) Fear of Negative Evaluation	r	0.191^**^	1				
		p	0.000					
	3) Acceptance of Power	r	0.106^*^	0.262^**^	1			
		p	0.023	0.000				
	4) Submission to Power	r	0.126^**^	0.427^**^	0.468^**^	1		
		p	0.007	0.000	0.000			
	5) Legitimization of Power	r	0.197^**^	0.137^**^	0.376^**^	0.333^**^	1	
		p	0.000	0.003	0.000	0.000		
	6) Instrumental Use of Power	r	0.224^**^	0.325^**^	0.388^**^	0.516^**^	0.329^**^	1
		p	0.000	0.000	0.000	0.000	0.000	
Surgical Sciences	1) Attitude Toward Medical Error	r	1					
		p						
	2) Fear of Negative Evaluation	r	0.156^*^	1				
		p	0.015					
	3) Acceptance of Power	r	0.137^*^	0.297^**^	1			
		p	0.032	0.000				
	4) Submission to Power	r	0.115	0.126^*^	0.423^**^	1		
		p	0.073	0.050	0.000			
	5) Legitimization of Power	r	0.271^**^	0.255^**^	0.345^**^	0.351^**^	1	
		p	0.000	0.000	0.000	0.000		
	6) Instrumental Use of Power	r	0.170^**^	0.172^**^	0.199^**^	0.456^**^	0.362^**^	1
		p	0.008	0.007	0.002	0.000	0.000	
All	1) Attitude Toward Medical Error	r	1					
		p						
	2) Fear of Negative Evaluation	r	0.184^**^	1				
		p	0.000					
	3) Acceptance of Power	r	0.116^**^	0.268^**^	1			
		p	0.002	0.000				
	4) Submission to Power	r	0.122^**^	0.322^**^	0.452^**^	1		
		p	0.001	0.000	0.000			
	5) Legitimization of Power	r	0.223^**^	0.169^**^	0.364^**^	0.340^**^	1	
		p	0.000	0.000	0.000	0.000		
	6) Instrumental Use of Power	r	0.209^**^	0.284^**^	0.315^**^	0.490^**^	0.334^**^	1
		p	0.000	0.000	0.000	0.000	0.000	

*p < 0.05; **p < 0.01; Pearson chi square

In **internal medicine specialties** (Fig. 1), medical error reporting attitudes were positively correlated with fear of negative evaluation (*r* = 0.191, *p* < 0.05) and all power distance dimensions, including acceptance of power (*r* = 0.106, *p* < 0.05), submitting to power (*r* = 0.126, *p* < 0.05), legitimization of power (*r* = 0.197, *p* < 0.05), and instrumental use of power (*r* = 0.224, *p* < 0.05). Fear of negative evaluation showed positive correlations with acceptance of power (*r* = 0.262, *p* < 0.05), submitting to power (*r* = 0.427, *p* < 0.05), legitimization of power (*r* = 0.137, *p* < 0.05), and instrumental use of power (*r* = 0.325, *p* < 0.05).

In **surgical specialties** (Fig. 2), medical error reporting attitudes were positively correlated with fear of negative evaluation (*r* = 0.156, *p* < 0.05), acceptance of power (*r* = 0.137, *p* < 0.05), legitimization of power (*r* = 0.272, *p* < 0.05), and instrumental use of power (*r* = 0.170, *p* < 0.05), whereas no significant association was observed with submitting to power (*r* = 0.115, *p* > 0.05). Fear of negative evaluation was positively correlated with acceptance of power (*r* = 0.297, *p* < 0.05), submitting to power (*r* = 0.126, *p* < 0.05), legitimization of power (*r* = 0.255, *p* < 0.05), and instrumental use of power (*r* = 0.172, *p* < 0.05).

Across the **overall sample** (Fig. 3), medical error reporting attitudes demonstrated weak but statistically significant positive correlations with fear of negative evaluation (*r* = 0.184, *p* < 0.05) and all power distance dimensions, including acceptance of power (*r* = 0.116, *p* < 0.05), submitting to power (*r* = 0.122, *p* < 0.05), legitimization of power (*r* = 0.223, *p* < 0.05), and instrumental use of power (*r* = 0.209, *p* < 0.05). Fear of negative evaluation was weakly to moderately correlated with acceptance of power (*r* = 0.268, *p* < 0.05), submitting to power (*r* = 0.322, *p* < 0.05), legitimization of power (*r* = 0.169, *p* < 0.05), and instrumental use of power (*r* = 0.284, *p* < 0.05).

### Mediation analysis of power distance dimensions effect the relationship between fear of negative evaluation and attitudes toward medical errors

**Fig. 4:**
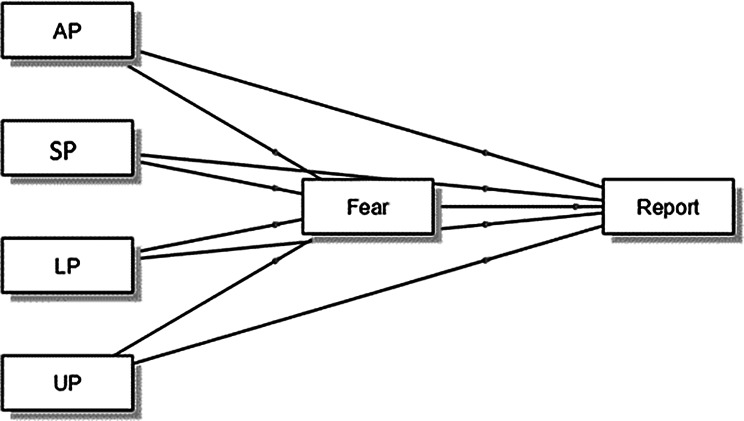
Pathways between attitude toward medical error reporting, fear of negative evaluation, and power distance dimensions; Acceptance of power (AP). Submitting to power (SP). Legitimization of power (LP). Using power instrumentally (UP)

Mediation analyses demonstrated that fear of negative evaluation played a significant intermediary role between specific power distance dimensions and attitudes toward medical error reporting (Table 4 and Fig. 4). Fear of negative evaluation significantly mediated the associations between instrumental use of power (β = 0.020, *p* = 0.013) and medical error reporting. Direct and total effects were significant for instrumental use of power (direct β = 0.137, *p* = 0.001; total β = 0.157, *p* < 0.001), indicating its dominant influence on reporting attitudes mediated by fear.

**Table 4:** Mediation analysis results showing direct, indirect, and total effects of power distance dimensions on medical error reporting by fear

Indirect and Total Effects								
				95% C.I. (a)				
**Type**	**Effect**	**Estimate**	**SE**	**Lower**	**Upper**	**β**	**z**	**p**
**Indirect**	**AP** ⇒ **Fear** ⇒ **Report**	0.00165	6.95e-4	2.84e-4	0.00301	0.01768	2.369	**0.018**
	**SP** ⇒ **Fear** ⇒ **Report**	0.00221	8.31e-4	5.80e-4	0.00384	0.02439	2.658	**0.008**
	**LP** ⇒ **Fear** ⇒ **Report**	1.74e-4	8.67e-4	-0.00153	0.00187	0.00103	0.200	0.841
	**UP** ⇒ **Fear** ⇒ **Report**	0.00152	6.14e-4	3.16e-4	0.00272	0.01955	2.474	**0.013**
**Component**	**AP** ⇒ **Fear**	0.30673	0.09255	0.12534	0.48813	0.13444	3.314	< 0.001
	**Fear** ⇒ **Report**	0.00537	0.00158	0.00226	0.00847	0.13150	3.387	< 0.001
	**SP** ⇒ **Fear**	0.41153	0.09601	0.22335	0.59970	0.18550	4.286	< 0.001
	**LP** ⇒ **Fear**	0.03238	0.16131	-0.28378	0.34854	0.00783	0.201	0.841
	**UP** ⇒ **Fear**	0.28311	0.07818	0.12989	0.43634	0.14865	3.621	< 0.001
**Direct**	**AP** ⇒ **Report**	-4.31e − 4	0.00392	-0.00812	0.00726	-0.00463	-0.110	0.913
	**SP** ⇒ **Report**	-0.00404	0.00409	-0.01206	0.00398	-0.04462	-0.987	0.323
	**LP** ⇒ **Report**	0.02903	0.00678	0.01573	0.04233	0.17214	4.279	< 0.001
	**UP** ⇒ **Report**	0.01067	0.00332	0.00417	0.01718	0.13734	3.216	0.001
**Total**	**AP** ⇒ **Report**	0.00121	0.00393	-0.00648	0.00891	0.01305	0.309	0.757
	**SP** ⇒ **Report**	-0.00183	0.00407	-0.00982	0.00615	-0.02023	-0.449	0.653
	**LP** ⇒ **Report**	0.02921	0.00684	0.01579	0.04262	0.17317	4.267	< 0.001
	**UP** ⇒ **Report**	0.01219	0.00332	0.00569	0.01869	0.15689	3.676	< 0.001

Note. Confidence intervals were computed using the standard (delta method). Betas are completely standardized effect sizes. Acceptance of power (AP). Submitting to power (SP). Legitimization of power (LP). Using power instrumentally (UP)

## Discussion

This multicenter study suggests that although exposure to medical errors was relatively common (51.9%), positive attitudes toward error reporting remained limited (4.5%), highlighting the potential role of perceived interpersonal risks and organizational norms in shaping reporting attitudes. By applying a mediation framework, the findings contribute to the patient safety and organizational culture literature by indicating that fear of negative evaluation may represent a psychological mechanism linking power distance dimensions with attitudes toward medical error reporting, underscoring its multilevel impact on communication and safety culture in healthcare [22].

This aligns with organizational theory describing power distance as accepted inequalities that shape work processes at multiple levels [23]. Our findings provide a possible psychological explanation for how hierarchical organizational structures may influence attitudes toward medical error reporting. In high-power-distance settings, feedback can be seen as disrespect or incompetence, making reputational harm a mechanism linking hierarchy to reduced transparency. A healthcare review revealed power distance themes across interpersonal and cultural levels, showing how fear processes are embedded in interactions (e.g., “don’t wake the senior,” “don’t ask questions”) [22].

Although the indirect effect size was modest, fear of negative evaluation emerged as a statistically significant intermediary factor linking instrumental use of power with attitudes toward medical error reporting. This finding may suggest that in environments where power is used strategically within hierarchical structures, healthcare professionals could perceive greater evaluative pressure, which may be associated with less favorable attitudes toward transparent error reporting despite formal patient safety policies. This fits the broader literature linking high power distance to constrained communication and reduced speaking up and helps explain why interventions that address only knowledge or reporting procedures often fail [24].

Cross-national evidence supports the relevance of these findings beyond the Turkish context in several ways. In Moroccan hospitals, professional hierarchies routinely override formal organizational structures: medical specialists occupy dominant positions, and power operates through negotiated informal mechanisms rather than strictly positional authority [25]. In Nigeria, professional control theories explain how medical professionals maintain dominant positions, creating structural barriers for other health professions [26]. Across these settings, the instrumental deployment of hierarchical dominance consistently cultivates fear of negative evaluation, undermines psychological safety, and suppresses transparent error reporting—patterns that align closely with the present study’s findings and reinforce the cross-cultural applicability of the proposed mediation model’s findings.

In comparison of professions in our study samples, nurses versus doctors demonstrated a lower tendency toward medical error reporting and lower levels of fear of negative evaluation. Nurses also exhibited higher scores across the power distance dimensions, with statistically significant differences in submission to power and the instrumental use of power. This finding suggests that nurses tend to represent a passive adaptation to authority driven by self-protective motives, through seeking to safeguard their professional standing within the hierarchy more than doctors. Consistent with Yang et al., nurses working in high power-distance settings perceive comparable barriers to error reporting. Cultural norms that emphasize hierarchical authority and face-saving intensify fears of negative consequences, indicating that reducing power distance and addressing face-saving concerns are critical for fostering psychologically safe, non-punitive reporting environments [27]. In a survey of 106 resident physicians from neurosurgery, orthopaedic surgery, emergency medicine, otolaryngology, neurology, obstetrics and gynaecology, paediatrics, and general surgery, perceived power distance negatively and leader inclusiveness positively predicted psychological safety, which in turn increased intentions to report adverse events. Psychological safety mediated the effects of both power distance and leader inclusiveness on reporting attitudes, emphasizing that adverse event reporting is shaped by organizational culture and hierarchical relationships rather than individual factors alone [28]. A large cross-national study across 14 European countries demonstrated that power distance was consistently and negatively associated with nurse-reported relations with physicians, indicating that hierarchical cultures undermine interprofessional collaboration [29]. In the study by Lim et al., physicians reported witnessing disruptive behaviors more frequently than nurses and perceived stronger associations with negative staff and patient outcomes [30]. This finding may partly explain the higher fear of negative evaluation observed among physicians relative to nurses in our study sample. Notably, married participants demonstrated higher acceptance and legitimization of power than single participants, which may reflect greater sensitivity to workplace stability and hierarchical structures among individuals with increased personal responsibilities. Future studies should further explore the relationship between marital status, risk perception, and attitudes toward authority in clinical settings.

Internal medicine departments reported higher fear of negative evaluation and greater instrumental use of power than surgical departments. Fear of negative evaluation also emerged as a significant intermediary factor in this association, supporting the importance of psychologically safe and non-punitive clinical environments. This aligns with Tigard’s ethical analysis, which argues that non-punitive environments are essential for meaningful error reconciliation [31]. Reported error prevalence estimates vary widely across studies due to differences in definitions and study designs [32], underscoring the importance of standardised, psychologically safe reporting systems. Evidence from surgical training shows burnout independently increases self-reported error rates [33], and job autonomy buffers burnout more strongly for clinicians with lower power distance orientations [34], reinforcing the view that reducing hierarchical instrumentalisation has cascading benefits for both safety culture and staff well-being.

Evidence from a multinational corporation across 39 countries demonstrates substantial cross-national variation in power distance, even within organisations sharing identical formal structures [35]. As reported in Section “Differences in attitudes toward medical errors and power distance dimensions” (Table 2), acceptance of power differed significantly by geographical region of childhood in the present study, with Marmara-raised participants showing higher scores than those from Southeastern Anatolia (*p* = 0.025). To our knowledge, this is the first study to examine childhood geography as a correlate of organisational power distance perceptions in a healthcare professional sample, positioning this as a novel contribution to the literature.

Participants raised in the Marmara region showed higher acceptance of power compared with those from Southeastern Anatolia, reflecting how socioeconomic development and early institutional exposure may shape deference to authority. Importantly, fear of negative evaluation and error reporting attitudes did not differ significantly by childhood region (Table 2), indicating that geographical background influences how hierarchy is internalised but does not directly predict willingness to report errors. Safety culture interventions should therefore target evaluative fear and reporting norms rather than treating childhood geography as a predictive risk factor for under-reporting.

### Limitations

This study has several limitations. First, its cross-sectional design precludes causal inference, and the temporal direction of the observed associations cannot be established. Second, all measures were self-reported and may therefore be subject to social desirability bias, particularly in hierarchical clinical environments. In addition, the study evaluated attitudes toward medical error reporting rather than objectively verified reporting behaviors. Although voluntary participation may have introduced selection bias, this risk was partially reduced by recruiting participants from five tertiary hospitals across both internal medicine and surgical departments. Finally, the observed effect sizes were generally small, and the practical significance of some statistically significant findings should therefore be interpreted cautiously.

## Conclusion

In conclusion, the findings suggest that organizational hierarchies may be associated with attitudes toward medical error reporting through interconnected psychological and organizational factors. Instrumental use of power and fear of negative evaluation were associated with less favorable reporting attitudes, while differences across professional roles and clinical divisions highlight the potential influence of workplace culture and hierarchical structures on openness toward transparent reporting practices. Uncertain results about error reporting tolerance in reporter’s clinic environment promotes self-censorship and discourages reporting. Consistent with WHO patient safety principles and the Just Culture framework [36], these findings support the need for institution-wide policies that promote psychologically safe, non-punitive reporting environments and reduce hierarchical barriers to transparent error reporting.

**Fig. 5:**
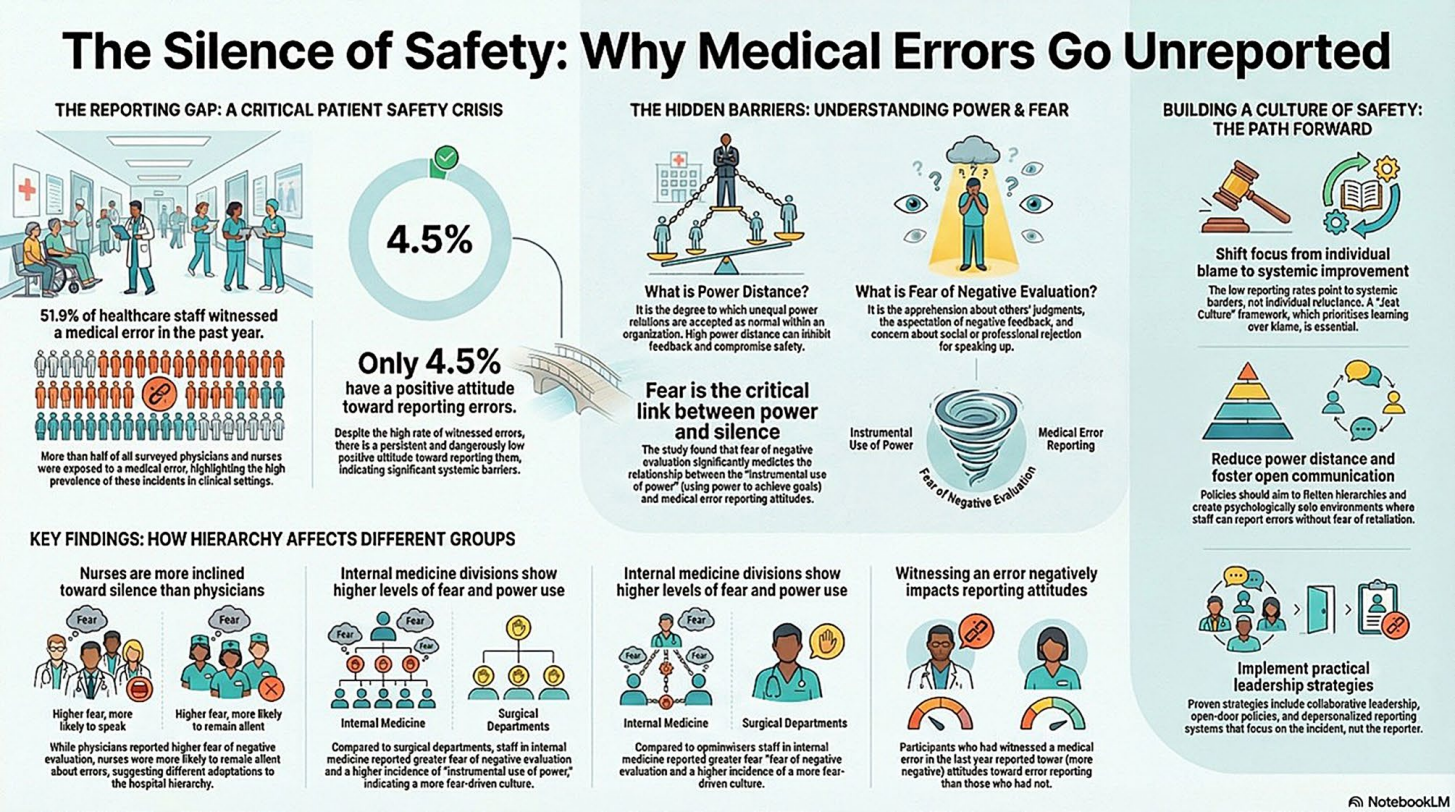
Visual absract of study

To address power distance in healthcare teams, strategies such as collaborative leadership, open-door communication, and nonpunitive reporting approaches have been recommended [24, 37]. From a health management perspective, reporting systems should prioritize learning and system improvement over individual blame (Graphic abstract in Fig. 5**)**.

## Data Availability

No datasets were generated or analysed during the current study.

## Declarations

### AI disclousure statement

During the preparation of this work, the authors used AI-assisted tools (Paperpal for grammar checking and language editing; ChatGPT-4 for assistance with literature search and thematic organisation of the discussion). ChatGPT was not used to generate, interpret, or analyse any study data; all quantitative analyses were performed exclusively using jamovi (version 2.7.11) and SPSS 25.0 by the study authors. ChatGPT was used solely to assist with identifying relevant literature and structuring argument flow in the discussion section. All AI-assisted content was critically reviewed, revised, and verified by the authors, who take full responsibility for the accuracy and integrity of the published work. After using this tool/service, the author(s) reviewed and edited the content as needed and take(s) full responsibility for the content of the published work.
